# The role of learning-walk related multisensory experience in rewiring visual circuits in the desert ant brain

**DOI:** 10.1007/s00359-022-01600-y

**Published:** 2022-12-09

**Authors:** Wolfgang Rössler, Robin Grob, Pauline N. Fleischmann

**Affiliations:** Behavioral Physiology and Sociobiology (Zoology II), Biocenter, University of Würzburg, 97074 Würzburg, Germany

**Keywords:** Central complex, Mushroom body, Multisensory navigation, Visual memory, Neuronal and synaptic plasticity

## Abstract

Efficient spatial orientation in the natural environment is crucial for the survival of most animal species. *Cataglyphis* desert ants possess excellent navigational skills. After far-ranging foraging excursions, the ants return to their inconspicuous nest entrance using celestial and panoramic cues. This review focuses on the question about how naïve ants acquire the necessary spatial information and adjust their visual compass systems. Naïve ants perform structured learning walks during their transition from the dark nest interior to foraging under bright sunlight. During initial learning walks, the ants perform rotational movements with nest-directed views using the earth’s magnetic field as an earthbound compass reference. Experimental manipulations demonstrate that specific sky compass cues trigger structural neuronal plasticity in visual circuits to integration centers in the central complex and mushroom bodies. During learning walks, rotation of the sky-polarization pattern is required for an increase in volume and synaptic complexes in both integration centers. In contrast, passive light exposure triggers light-spectrum (especially UV light) dependent changes in synaptic complexes upstream of the central complex. We discuss a multisensory circuit model in the ant brain for pathways mediating structural neuroplasticity at different levels following passive light exposure and multisensory experience during the performance of learning walks.

## Introduction

Many insect species, particularly social insects like ants or social bees and wasps, heavily depend on navigational skills for food provisioning of their brood. As offspring is raised in a common nest, the ants search for profitable food locations and then return to their nest (central place foraging). Desert ants of the genus *Cataglyphis* are excellent experimental models to study the mechanisms underlying insect navigation (Wehner 2008, 2020). These thermophilic ants live under mostly harsh conditions. *Cataglyphis* ants leave their underground nest even during the hottest times of the day for far-ranging food searches. These excursions may extend to walking distances of up to 1,500 m and distances of 350 m or more away from the nest (e.g. Huber and Knaden 2015; Ronacher 2008). *Cataglyphis* species inhabiting featureless desert environments employ path integration as their main navigational routine to find their way back to the nest (Knaden and Graham 2016; Ronacher 2008; Wehner 2020). Foraging ants use the position of the sun and the associated sky-polarization cues to determine the directions of all path segments during the outbound walks, while a step integrator (Wittlinger et al. 2006, 2007) estimates the distances used to calculate a home vector that encodes both the direction and distance back to the nest entrance. Especially *Cataglyphis* ant species living in more cluttered environments use the panoramic scenery and landmarks as additional visual cues (Fleischmann et al. 2017; Fleischmann et al. 2018a; Huber and Knaden 2015; Wehner 2020; for a review see Cheng et al. 2014).

The navigational routines of mainly visually guided foraging ants have been characterized in much detail at the level of behavior and generated valuable models (for an excellent and extensive recent review see Wehner 2020). The question of how naïve ants acquire their navigational information during so-called learning walks at the beginning of their foraging careers, particularly the underlying neuronal processes, have gained attention only more recently (for reviews: Grob et al. 2019; Rössler 2019; Wehner and Rössler 2013). Path integration, visual learning, and learning walks are widespread in other species of ants including other desert-ant species like *Ocymyrmex* or *Melophorus* and non-desert ant species like *Myrmecia* bull ants and *Formica* wood ants (e.g. Deeti and Cheng 2021; Jayatilaka et al. 2018; Müller and Wehner 2010; Nicholson et al. 1999; Wystrach et al. 2014a, b).

In the present review, we integrate results from studies on learning walks with a special focus on *Cataglyphis* ants. We provide a synthesis of the knowledge gained on neuronal plasticity (neuroplasticity) in visual circuits of the ant’s brain triggered by multisensory experience during learning walks that are performed during a sensitive period before the onset of foraging behavior. Most studies on neuroplasticity have been done in *Cataglyphis* ants. We complement these by looking at select neuroanatomical, neurophysiological, and behavioral studies on multisensory pathways in the brain of other insects. This synthesis aims at understanding the neuronal circuits mediating multisensory experience during learning walks and at stimulating future studies to unravel the neuronal mechanisms underlying the striking flexibility of navigational systems in the brain of these remarkable navigators.

## Multisensory experience during the performance of learning-walks

*Cataglyphis* foragers use path integration to return to their nest on the shortest distance possible by integrating directional (compass) and distance information. The resulting home vector leads the ants back to their nest entrance along an almost straight path (Wehner 2020). A recent study manipulated path integration memory by experimental cooling of ants that had built up a full home vector after finding food (Pisokas et al. 2022). In this study, the behavioral analyses of homing trajectories were combined with computer simulations of homing strategies based on different path-integration memory models. The results suggest that path integration memory is most likely stored in a redundant Cartesian coordinate system in the ant’s brain. However, despite such an efficient mechanism, the underlying neuronal processes are prone to cumulative errors, especially over long distances. To compensate for these errors and to optimize homing, the ants, whenever available, use panoramic sceneries, the skyline, and landmarks as additional visual guidance cues (e.g., Wehner et al. 2016; Wystrach et al. 2015).

How do naïve ants that leave their nest for the first time acquire the necessary information about relevant sky-compass cues and the panoramic scenery around the nest entrance to ensure efficient homing? Using a sun-based compass for navigation comprises additional challenges (for the definition of navigation see Grob et al. 2021c). How do the ants calibrate their sky compass system? The ants use the azimuthal position of the sun and associated sky-polarization patterns as compass cues. However, the azimuthal course of the sun over the day (solar ephemeris) is a non-linear function that depends on the time of the year and the geographical position. Consequently, an internal representation of the solar ephemeris cannot be inherited genetically, and the ants somehow need to calibrate their internal compass systems with the function representing the azimuthal path of the sun (Wehner 2020; Wehner and Lanfranconi 1981; Wehner and Müller 1993). How do naïve ants acquire this function and what is used as the earthbound compass reference during learning the relevant visual information? What are the neuroplastic changes underlying visual learning and long-term memory formation before the ants are heading out on their first foraging trips (Rössler 2019)?

The individual life history of *Cataglyphis* ants comprises a period of about 4 weeks with varying tasks inside the darkness of the underground nest followed by an outdoor foraging period of on average 7 days in bright sunlight when the ants actively search for food (Schmid-Hempel and Schmid-Hempel 1984). Because of this drastic transition and striking differences in the associated behavioral phenotypes (polyethism), *Cataglyphis* ants are ideal experimental models for investigating the neuronal mechanisms underlying this behavioral plasticity (Rössler 2019). The interior-exterior transition will be the focus of the following chapters.

**Fig. 1:**
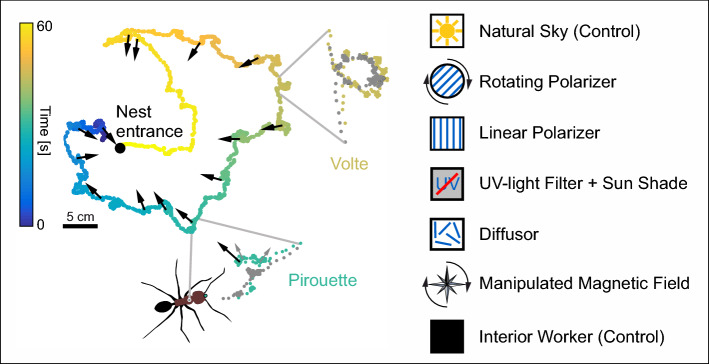
Learning walks and their experimental manipulation in *Cataglyphis* ants. Left: Typical example of an elaborated learning walk in *Cataglyphis nodus* (modified from Grob et al. 2019; data from Fleischmann et al. 2017). The ants walk in small loops around their nest entrance (black dot). During repeated interruptions of their forward movements, the ants perform pirouettes and look back (black arrows) to the nest entrance. Time is color coded. Insets lower and upper right. The ants perform rotations (pirouettes) about their own vertical body axes (lower right) while making brief stops (> 100 ms) (arrows). During the longest stopping phase (black arrow), their views are directed towards the nest entrance. In addition to pirouettes, *C. nodus* ants perform small, walked circles (voltes) with only very rare stops. For both rotations, the tracking positions of the mandibles (green) and the thorax (gray) are indicated (modified from Fleischmann et al. 2017). Right Symbols depicting manipulations that have been applied during learning-walk experiments and during passive light exposure of the ants. From top to bottom: under natural skylight (control); under a rotating linear polarizer; under a stationary linear polarizer; under UV block and with the sun shaded; under a diffusor; with a manipulated magnetic field; unexperienced workers from the dark nest interior that have not yet performed learning walks (control)

Behavioral studies over recent years revealed important insights into so-called learning walks (also termed exploration or orientation walks) that naïve ants perform before they head out on their first foraging excursions (for recent reviews on learning walks: Fleischmann et al. 2020a; Freas et al. 2019; Zeil and Fleischmann 2019). To understand how naïve ants acquire the relevant visual information, quantitative analyses of learning-walk behavior at high spatial and temporal resolution in the natural habitat was a crucial step*.* Naïve ants perform structured learning walks comprising sequences of loops that explore different sectors around the nest entrance (Fleischmann et al. 2016, 2017; Stieb et al. 2012; Wehner et al. 2004) (Fig. 1). The ants perform these behavioral routines in an extending radius around the nest entrance over a period of 2–3 days. During that time, they do not collect any food items (Fleischmann et al. 2016). High-resolution video analyses revealed that the ants frequently stop their forward movements to perform rotational elements (pirouettes) interrupted by 100–200 ms stops. During the longest stop in a pirouette, the ants look back in the direction of the nest entrance. Similar findings were made earlier in a study on re-learning walks in *Ocymyrmex a*nts (Müller and Wehner 2010). As the nest entrance is not visible from the ants’ perspectives, the ants must use path integration for the alignments of their body axis during these repeated turn-back behaviors.

The highly structured behavior during learning walks suggests that ants and other insects like wasps (during their learning flights) systematically acquire and memorize nest-directed panoramic views around the nest entrance (Graham et al. 2010; Zeil 2012). However, the acquisition of nest-related views may vary across species. Furthermore, recent work on wasps suggests that they do not pause when acquiring views 45° left and right from the nest entrance (Stürzl et al. 2016). Another study on ants indicates that views are memorized in other directions than the nest direction (Wystrach et al. 2020). In addition to acquiring panoramic snapshot memories, the ants most likely calibrate their internal solar ephemeris representation during learning walks (Grob et al. 2019). Evidence supporting this assumption most recently came from a study showing that only learning walks under a rotating sky polarization pattern resulted in plastic neuronal changes in visual integration centers of the ant’s brain (Grob et al. 2022) (see details in the following chapters). Furthermore, displacement experiments show that *Cataglyphis* ants need at least 2 days and a minimum distance of 0.5 m from the nest entrance for the proper performance of learning walks to memorize panoramic cues for successful homing (Fleischmann et al. 2016, 2018a). These findings are supported by earlier studies in *Melophorus* desert ants (Wystrach et al. 2012).

What provides the compass reference for path integration and panoramic snapshot learning during initial (or naïve) learning walks, at a time when the celestial compass has not yet been calibrated? To ask whether naïve ants nevertheless use the sun as a compass for path integration during their short learning walks, different filter settings above the nest entrance were used to manipulate relevant skylight cues (Grob et al. 2017). With important celestial cues blocked (sun position, sky polarization pattern, UV light), the ants still perform stops with accurately aligned goal-directed views during learning-walk pirouettes. Although it had been shown that spectral and light intensity gradients can also be used for a celestial compass (Wehner 1997; Wystrach et al. 2014a, b), the ants might as well use other compass cues at this early stage. The earth’s magnetic field came into focus as a candidate for an alternative compass used during initial learning walks. Under experimental conditions using disarray of the earth’s magnetic field by a circular flat coil or elimination of the horizontal component of the earth’s magnetic field by a Helmholtz coil setup, the ants gazed into random directions even with all natural celestial cues accessible (Fleischmann et al. 2018b). Moreover, systematic rotation of the horizontal component of the earth’s magnetic field resulted in gazes towards a fictive nest entrance rotated by the same angle as the magnetic field. This provided the first unambiguous evidence that an insect can use the earth’s magnetic field as a compass cue that is both necessary and sufficient for path integration. This also suggests that the geomagnetic field provides the earthbound compass reference for path integration during initial learning walks. Consequently, the magnetic sense of *Cataglyphis* ants provides the compass reference during view-based learning of the visual panorama and can potentially be used to calibrate the celestial compass systems. Finally, the unambiguous role of the magnetic sense in path integration renders *Cataglyphis* ants as a highly promising experimental model for the study of magnetoreception in an insect (Fleischmann et al. 2020b). Compared to the clear-cut results in *Cataglyphis*, the use of the earth’s magnetic field for directional orientation in other insect species, so far, was less distinct or only evident in combination with other cues (e.g. Dreyer et al. 2018; see reviews in Fleischmann et al. 2020b; Wajnberg et al. 2010). Interestingly, experienced *Cataglyphis* foragers still sense the magnetic field but the function of magnetic field information in orientation is different from the one in naïve ants. This was recently shown by using magnetic manipulations in the natural habitat and during so-called re-learning walks of foragers (Fleischmann et al. 2022).

Overall, the behavioral results in *Cataglyphis* suggest that during their first learning walks the ants experience panoramic snapshot views, skylight compass cues, and directional cues provided by the earth’s magnetic field as important sensory information. In the following chapters, we will have a closer look into the ants’ brains and the associated sensory pathways to then focus on neuroplasticity in visual pathways triggered during different phases of the interior-exterior transition.

## The brain of *Cataglyphis* and sensory pathways associated with navigation

**Fig. 2:**
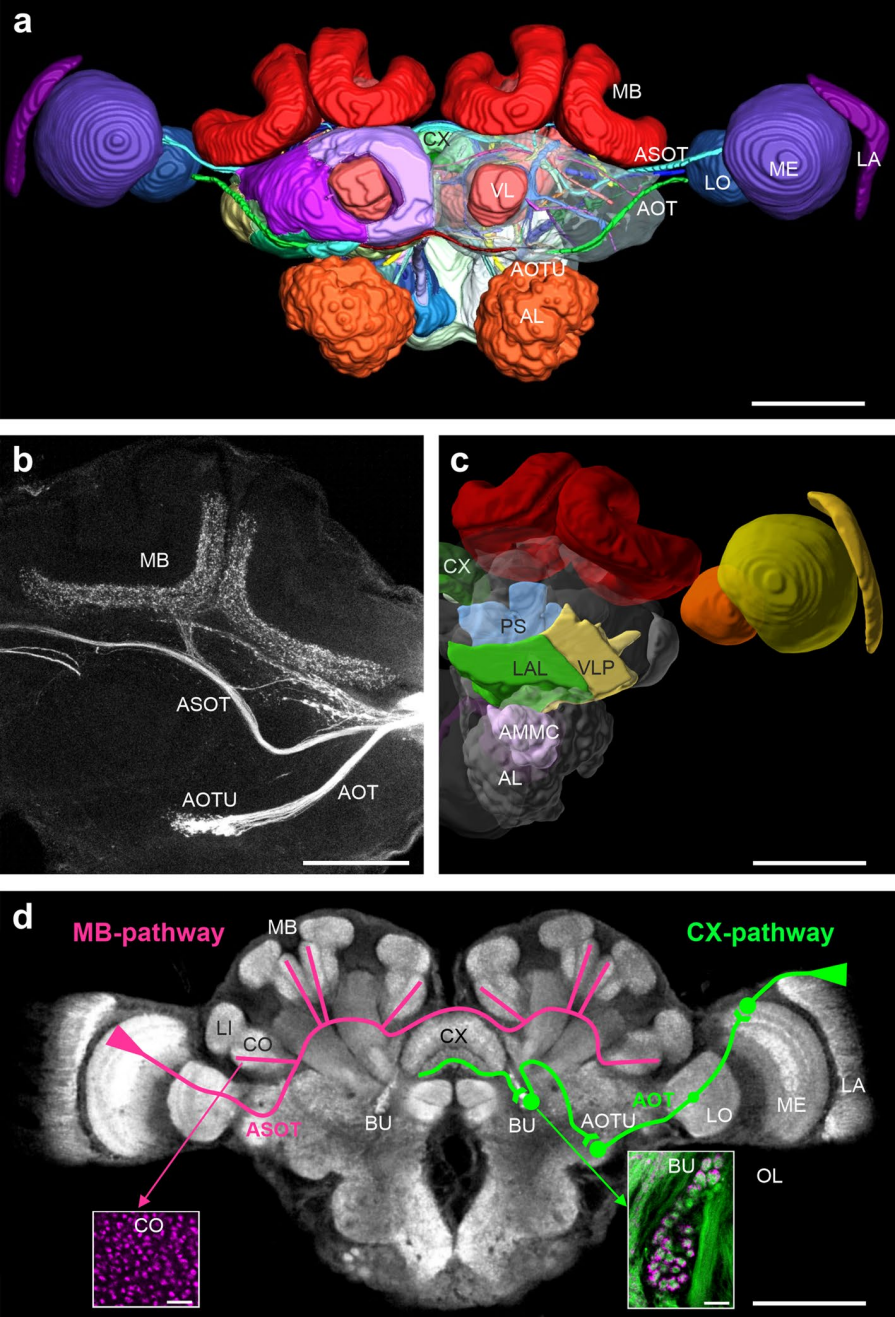
The brain of *Cataglyphis nodus* with visual pathways into different brain neuropils. **a** Overview of a 3D-reconstruction of the brain with color-coded major neuropils and partly exposed neuronal tracts (from Habenstein et al. 2020—modified by Jens Habenstein). Scale bar is 200 µm. **b** Confocal images of original fluorescent tracings (rhodamine dextran with biotin, microruby) of the anterior optic tract (AOT) and the anterior superior optic tract (ASOT) (modified from Grob et al. 2017). Scale bar is 100 µm. **c** 3D-reconstruction of the left brain hemisphere highlighting neuropils that receive input from afferent sensory projections of Johnston's organ in *C. nodus* (modified from Habenstein et al. 2020 using Insect Brain Database; https://www.insectbraindb.org/). Scale bar is 200 µm. **d** 2D confocal projection of the brain of *C. nodus* labeled with an antibody to synapsin with the schematic courses of the two major visual tracts (modified from Grob et al. 2017 and Rössler 2019). The anterior pathway with the AOT is labeled in green, the ASOT in magenta. Scale bar is 200µm. Left inset Large synaptic boutons of visual projection neurons in the mushroom-body (MB) collar (anti synapsin immunoreactivity labeled in magenta). Right inset Anti synapsin- (magenta) and phalloidin-labeled (green) microglomerular synaptic complexes in the bulb of the lateral complex. Scale bar in both insets is 10 µm. *AL* antennal lobe, *AMMC* antennal mechanosensory and motor center, *AOT* anterior optic tract, *AOTU* anterior optic tubercle, *ASOT* anterior superior optic tract, *BU* bulb, *CO* collar, *CX* central complex, *LA* lamina, *LAL* lateral accessory lobe, *LI* lip, *LO* lobula, *MB* mushroom body, *ME* medulla, *OL* optic lobe, *PS* posterior slope, *VL* vertical lobe, *VLP* ventrolateral protocerebrum

How does the relatively small brain of *Cataglyphis* ants process and memorize multisensory navigational information during learning walks? Where in the brain are sky-compass cues and local panoramic views computed and stored? How is visual information integrated with input from a magnetic compass and from other sensory modalities? While the first two questions can be addressed more easily, the third question, particularly regarding the magnetic sense, is more difficult to answer. Recently a comprehensive 3D atlas of the entire brain of *Cataglyphis nodus* has been published and provides a solid ground for neuroanatomical analyses (Habenstein et al. 2020) (Fig. 2a, c). For comparability, the nomenclature for individual neuropils was adapted to the unified nomenclature applied to the brain atlas in the fly, *Drosophila melanogaster* (Ito et al. 2014). The 3D brain atlas in *Cataglyphis nodus* workers revealed 33 distinct brain neuropils and 30 connecting fiber tracts including six visual fiber tracts between the optic lobes and the central brain (Habenstein et al. 2020). The *Cataglyphis* brain atlas is accessible in 3D on the Insect Brain Database website (https://www.insectbraindb.org/) (Habenstein et al. 2020; Heinze et al. 2021). Neuroanatomical analyses in another study revealed differences in 3D structures and volumes of brain compartments between the two female castes (workers and queens) and males (Grob et al. 2021b), most likely reflecting differences in their lifestyles and navigational skills.

In addition to the neuroanatomical studies, analyses of neuropeptides and their localization in the *Cataglyphis* brain have recently been performed. For this, transcriptomal analyses were combined with mass spectrometry detection and localization to reveal the most comprehensive neuropeptidome that has been obtained from an ant’s brain so far (Habenstein et al. 2021a, 2021b; Schmitt et al. 2017). Both the neuroanatomical and neuropeptidome databases provide rich sources for future structure–function analyses in the brain of *Cataglyphis* ants, for example, to detect life-stage dependent changes in brain neuropils and neuromodulators, and for comparison of those attributes with other ant species*.*

## Visual pathways from the optic lobes

Where in the ant’s brain are sky compass cues and panoramic information processed and stored? Early behavioral manipulations have already indicated that local panoramic information is stored as long-lasting (lifetime) memories, whereas path integration information expresses a faster memory decay. This early discovery was already highly suggestive of two distinct channels for visual information transfer (Ziegler and Wehner 1997). Neuroanatomical studies revealed the anatomical details of two separate major visual pathways leading from the optic lobes to two high-order visual integration centers in *Cataglyphis’* brain, the mushroom bodies (MB) and the central complex (CX) (Grob et al. 2017; Rössler 2019; Schmitt et al. 2016) (Fig. 2b, d). The presence of a visual pathway to the MB in ants was first shown by Gronenberg and Hölldober (1999) and Gronenberg (2008). In *Cataglyphis* these neuronal pathways were traced using fluorescent dye injections, confocal imaging, and 3D reconstructions. Recently, Habenstein et al. (2020) mapped four additional tracts and commissures from the optic lobes to the central brain in *Cataglyphis nodus*. In the following, we outline the two major visual circuits from the optic lobes to the CX and MB in *Cataglyphis* and compare these with the conditions in other insect species.

One pathway starts from the dorsal rim area (the polarization-sensitive part of the compound eye) and proceeds via the lamina, medulla, and lobula along the anterior optic tract (AOT) to the anterior optic tubercle (Schmitt et al. 2016) (Fig. 2b, d). From there, neurons project to the bulbs of the lateral complex where they form exceptionally large microglomerular synaptic complexes that are synaptically connected with the dendrites of tangential neurons which project to the lower unit of the central body, a subdivision of the CX (Grob et al. 2017, 2019; Schmitt et al. 2016). Accordingly, this pathway was termed CX pathway. Physiological and neuroanatomical studies in the locust, bee, dung beetle and monarch butterfly combined with modeling approaches show that this highly conserved pathway to the CX integrates sky-compass information and receives information about movement, the two parameters required for path integration (e.g. Heinze and Homberg 2007; Heinze and Reppert 2011; Held et al. 2016; Homberg et al. 2011; Kaiser et al. 2022; Stone et al. 2017; Webb and Wystrach 2016).

The second pathway leads from the medulla of the optic lobes via the anterior superior optic tract (ASOT) without any further relay to the visual compartments (collar) of the MB and was termed MB pathway (Ehmer and Gronenberg 2002; Grob et al. 2017, 2019) (Fig. 2b, d). Phylogenetic comparison within the Hymenoptera suggests that this prominent visual pathway to the MBs has evolved in the higher Hymenoptera, particularly parasitoid and social Hymenoptera (Farris and Schulmeister 2011). Two recent ablation studies in ants, one using injections of local anesthetics at the level of the visual projection neuron input in *Formica* wood ants (Buehlmann et al. 2020), and another study in *Myrmecia* bull ants using local applications of anesthetics to the vertical lobes, major output structures of the MBs (Kamhi et al. 2020) came to the coincident conclusion that the MBs are required for panoramic landmark orientation during homing. In the MB collar of the honeybee and *Cataglyphis*, visual projection neurons from the medulla of the optic lobe form very large presynaptic boutons comprising many (~ 60) active zones (synaptic contacts) connected to numerous dendrites of postsynaptic Kenyon cells (the MB intrinsic neurons) forming microglomerular synaptic complexes (Groh et al. 2012; Stieb et al. 2010, 2012).

While both the CX and MB visual pathways comprise large synaptic complexes at the input of their target neuropils including many postsynaptic contacts, there is a significant difference between the two circuits (Fig. 2d). Whereas the total number of microglomerular synapses in the MB collars of both brain hemispheres was estimated at ~ 400,000 in *Cataglyphis*, only ~ 100 microglomerular synapses are implemented in the bulbs of both hemispheres, the terminal synaptic relay stations in the CX pathway (reviewed in Grob et al. 2019; Rössler 2019). The enormously large and highly divergent synaptic matrix in the MB collar stands in contrast to the CX pathway converging on only ~ 100 synaptic complexes on both sides. Increasing evidence from physiological studies supports the function of the CX pathway as the crucial sky-compass pathway transferring relevant skylight cues including light polarization in the UV range, sun position, and UV-green chromatic gradients (el Jundi et al. 2014; Homberg et al. 2011). For the MB pathway, the enormous capacity of the MB collars for the storage of view-based panoramic information is further supported by modeling studies (Ardin et al. 2016; Peng and Chittka 2017) and by the above-mentioned ablation studies.

## Sensory projections from Johnston’s organ

The important role of magnetic information for goal-directed views during learning walks of *Cataglyphis nodus* (Fleischmann et al. 2018b) provokes the question of how magnetic information is integrated with input from the two visual pathways described above. The hymenopteran antenna has repeatedly been proposed as a promising candidate for the potential location of a magnetic sensor (reviewed in Fleischmann et al. 2020b; Wajnberg et al. 2010; and study by de Oliveira et al. 2010) with Johnston’s organ (JO) representing a potential candidate. The JO is located at the base of the insect antenna and was shown to function primarily as a mechanosensory organ with highly sensitive chordotonal sensilla inserted into a delicate membrane at the joint between the antennal pedicellus and flagellum (for a review see Yack 2004). The number of sensilla varies between species and depends on the main function of the organ. In general, the JO serves the detection of antennal deflections caused by wind, gravity, vibration (air- and substrate-borne) or touch (Kamikouchi et al. 2009). Interestingly, during learning walks, *Cataglyphis* ants perform very characteristic antennal movements (Wehner et al. 2004, and own unpublished observations), which may suggest that specific antennal movement patterns are necessary to receive the magnetic information in an active sensing process (Fleischmann et al. 2020b).

A recent anatomical study mapped the sensilla and associated sensory structures (scolopidia) of the JO in the antenna of *Cataglyphis nodus* workers, queens, and males (Grob et al. 2021a). In workers, about 40 scolopidia, each comprising three sensory neurons, project axons to the antennal mechanosensory and motor center and extend sensory afferents to neuropils in the posterior brain including the saddle, ventrolateral protocerebrum, ventral complex, and the posterior slope (Grob et al. 2021a; Habenstein et al. 2020). The overall architecture of the JO in *Cataglyphis* shows many similarities with the JO in the honeybee (Ai et al. 2007). Most interestingly, in both *Cataglyphis* and the honeybee, the posterior slope also receives projections from interneurons of the ocelli that are in close apposition with sensory terminals from JO afferents. In addition, in the ventrolateral protocerebrum, ventral complex, and posterior slope neuronal projections from the optic lobes via the posterior and inferior optic tracts converge with sensory afferents from the JO (Grob et al. 2021a; Habenstein et al. 2020). Connectome studies in *Drosophila* (Scheffer et al. 2020) revealed that visual input from the optic lobe in these regions is preferentially from the lobula. Importantly, the posterior slope together with the lateral accessory lobe and ventrolateral protocerebrum contain post-synaptic compartments of descending neurons relaying motor commands to the ventral nerve cord (Currier and Nagel 2020; Hsu and Bhandawat 2016; Namiki et al. 2018a). This classifies these neuropils as premotor centers. In *Drosophila*, many descending neurons are bimodal and respond to mechanosensory and visual input (Namiki et al. 2018b).

Before we draw further conclusions on the role of multisensory convergences in these neuropils in the ant’s central brain, the next chapter reviews the results from experimental manipulations of sensory input during the performance of learning walks in the natural habitat, particularly their consequences for neuroplastic changes along the two visual pathways described above.

## The role of multisensory experience in rewiring visual circuits

Recent studies have applied various manipulations of sensory input during learning walks in the natural habitat and subsequently screened the brains of the experimental ants for neuroplastic changes in visual circuits along the CX and MB pathways (Grob et al. 2019). For a behavioral readout, high-resolution video analyses of the pirouetting behavior with different stopping phases during learning walks were used as a reliable tool to test the ants’ information about the direction of the nest entrance and as an indicator of their path-integration abilities under the various experimental conditions (Fleischmann et al. 2017, 2018b, 2022; Grob et al. 2017). Subsequently in the laboratory, structural neuroplasticity along the CX and MB visual pathways served as a readout (or measure) for the role of sensory experience in triggering neuroplastic changes or calibrations in visual circuits (Grob et al. 2017, 2019, 2022; Schmitt et al. 2016; Stieb et al. 2010, 2012). Structural neuroplasticity was quantified either as changes in neuropil volume (CX and MB) or as variations in the number of synaptic complexes (MBs and bulbs of the lateral complex).

Before we bring together the outcome of these studies, let us first consider the nature of the different types of visual stimulation by differentiating two forms of experience—light exposure in the absence of learning-walk behavior (in the following termed “light exposure”) and learning-related visual experience during learning walks (in the following termed “learning walk”). Naïve ants experience light for the first time when they reach a point in their natural ontogeny where they approach the nest entrance for the first time from the inside of the nest and when ants, for example during nest-building activities, perform so-called short digging walks to expel material from the interior of the nest to the outside (Fleischmann et al. 2017; Wehner 2020; Zeil and Fleischmann 2019). In contrast, view-based sensory perception while performing pirouettes with goal-directed stops during learning walks may be regarded as an active learning process, and therefore, best characterized as learning-related experience. In addition to visual cues, the behavioral analyses have shown that the ants use the earth’s magnetic field as a compass reference for path integration during initial learning walks. If we assume that path integration combined with the experience of a nest-directed view, the homing direction, may function as an internal reward, we can assign this type of learning to a form of associative (Hebbian) learning and plasticity. Contrary to this, passive light exposure during an experimental situation where naïve ants do not (yet) perform leaning walks can be considered as non-associative sensory exposure. Consequently, the two types of experimental conditions for visual input were classified as light-exposure and learning-walk related visual experience. However, we must keep in mind that under natural conditions, the first combined experience of skylight and magnetic field may already happen during digging walks, or even earlier, when the ants sit in the nest entrance for the first time. Therefore, both behaviors may comprise transitions between the two forms of sensory experience.

## Structural neuroplasticity in visual pathways triggered by first light exposure

**Fig. 3:**
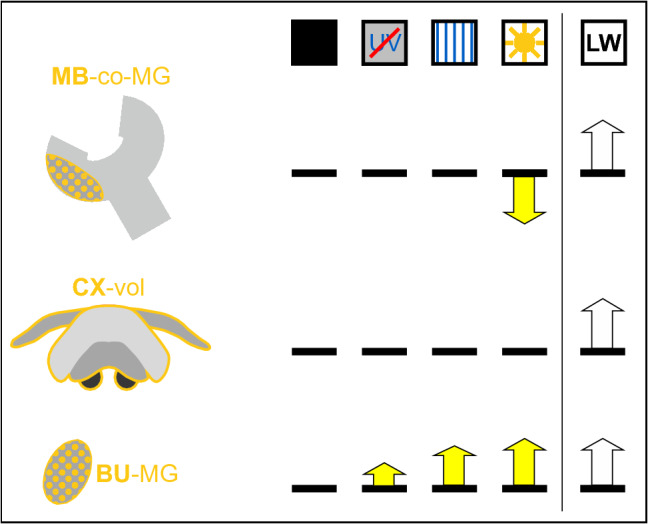
Summary of results on neuronal plasticity following passive light-exposure experiments in *Cataglyphis* ants. The top row depicts the different manipulations (the different symbols are explained in Fig. 1). The following rows summarize changes in the different neuropils. Arrows pointing upwards indicate significant increase in the number of synaptic complexes within neuropils or in the volumes of neuropils, arrows pointing downwards indicate respective significant decreases compared to the dark control conditions. No arrow means no significant change. The first row shows changes in the number of microglomerular synaptic complexes (microglomeruli) in the mushroom body (MB) collar (MB-co-MG). The second row summarizes changes in the volume of the central complex (CX-vol) under the various conditions. The bottom row shows changes in the numbers of microglomerular synaptic complexes in the bulb (BU-MG). For comparison, the column on the very right with light arrows depicts respective changes after the active performance of learning walks (LW) under natural skylight. See text for further details. Based on data from (Grob et al. 2017, 2022; Schmitt et al. 2016; Stieb et al. 2010, 2012)

Effects of light exposure have been tested in young ants that had not yet been outside the dark nest. When young *Cataglyphis* ants were precociously exposed to light, microglomerular synaptic complexes in both the CX and MB visual pathways exhibited structural plasticity (Schmitt et al. 2016; Stieb et al. 2010, 2012) (Fig. 3). Repeated application of 45 min light pulses over a period of four to five days resulted in a decrease in the number of microglomeruli in the visual compartments of the MB calyx, an indication of synaptic pruning. Interestingly, in the MB collar such light-exposure dependent synaptic changes could still be triggered in six- and twelve-month old ants that had been kept in constant darkness. This suggests that this type of plasticity adapts visual processing to different light conditions throughout the entire lifespan of the ants (Stieb et al. 2010). Similarly, precocious visual exposure in honeybees (Scholl et al. 2014) and olfactory sensory exposure to diverse plant odors in leaf-cutting ants over three to five days (Falibene et al. 2015) resulted in a decrease in the numbers of microglomeruli (pruning) in the MB collar and lip, respectively. Light exposure was also shown to induce changes in the expression of neuroplasticity-related genes in the honeybee (Becker et al. 2016).

Contrary to a decrease of microglomeruli in the MB collar following light exposure, the number of microglomerular synaptic complexes in the bulbs of the CX pathway showed an increase by up to 30% after first light exposure, even when applied to one-day old ants (Grob et al. 2022; Schmitt et al. 2016) (Fig. 3). At a first glance, the diverging effects on microglomerular synapses in the two pathways look contradictory. The reason for this difference likely resides in the function, particularly different neurotransmitter systems involved in the respective neuronal circuits. In both cases, structural synaptic plasticity was observed in microglomerular synaptic complexes comprising a large presynaptic bouton and numerous postsynaptic profiles (Rössler 2019). While visual projection neurons in the MB collar in *Cataglyphis* are most likely cholinergic and excitatory, tangential neurons that relay the information from bulb microglomeruli to the lower unit (ellipsoid body) of the central body in the CX are GABAergic (Schmitt et al. 2016). This feature is highly conserved in other insects (e.g. locust, Träger et al. 2008, and *Drosophila,* Seelig and Jayaraman 2015). If we consider the structural synaptic changes following first exposure to bright sunlight as a form of homeostatic plasticity that helps to maintain neuronal activity in a dynamic range, we can expect a reduction of excitatory synapses in the MB collars (synaptic pruning) and an increase of inhibitory (GABAergic) synapses in the bulb at the entrance to the CX. However, the exact function of this plasticity at the input remains to be determined as neuronal activity in both the MB and CX is also modulated through internal inhibitory feedback. Normalization of activity in the MB calyx is achieved through a set of GABAergic feedback neurons in the honeybee and the APL neuron in *Drosophila* (Grünewald 1999; Haehnel and Menzel 2012; Prisco et al. 2021). Normalization of activity in the ellipsoid body (input of the CX from the bulb) is controlled by CX local inhibition (Kim et al. 2017). A possible explanation might be that the intrinsic inhibitory circuits act on a smaller dynamic range compared to the changes at the input synapses, which may be required during the drastic changes in sensory input during the interior-exterior transition. Future synaptic connectivity studies at the ultrastructural level, especially of convergence-divergence ratios, are necessary to further determine the role of structural synaptic changes in both circuits in homeostatic plasticity.

Interestingly, the increase of microglomerular synaptic complexes in the bulbs of the CX pathway is light-quality dependent and especially sensitive to the presence of UV light (Grob et al. 2022; Schmitt et al. 2016). No structural changes were found in both the MB and CX pathways when artificially dark-kept ants were passively exposed to either a static or moving pattern of polarized light, even with the UV spectrum included (Grob et al. 2022). Similarly, no changes were found after passive light exposure regarding the overall volume of the MB calyx and CX neuropils. This shows that one or three days of passive light exposure were not sufficient to induce a decrease (pruning) of synaptic complexes in the MB collar (Stieb et al. 2012; Grob et al. 2022). We conclude that passive exposure to skylight over more than three days triggers a form of most likely homeostatic synaptic plasticity at the input to the CX and MB. In the CX pathway, the degree of change depends on the chromatic composition of the light stimulus.

## Structural neuroplasticity in visual pathways triggered by learning walks

**Fig. 4:**
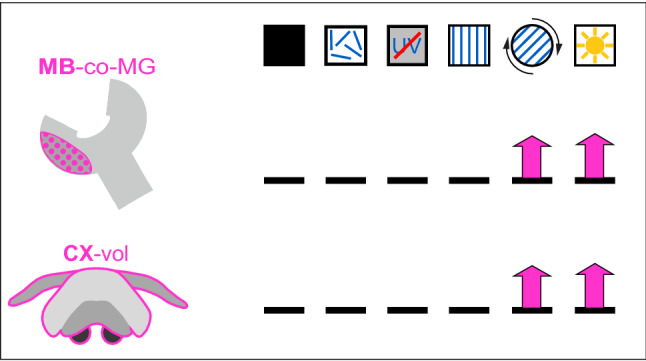
Summary of results on neuronal plasticity following the active performance of learning walks in *Cataglyphis* ants under various conditions. The top row depicts the different manipulations (the different symbols are explained in Fig. 1). The following rows summarize changes in the different neuropils. Arrows pointing upwards indicate significant increase in the numbers of synaptic complexes or neuropil volumes, arrows pointing downwards indicate a respective significant decrease compared to the dark control conditions. No arrow means no significant change. The first row shows changes in the number of microglomerular synaptic complexes in the mushroom-body calyx collar (MB-co-MG) after the various treatments. Only learning walks under natural skylight and under a rotating linear polarizer trigger a significant increase in the numbers of MB-co-MG. The second row shows changes in the central complex volume (CX-vol) following learning walks under the various treatments. Like effects in the mushroom bodies, the volume of the CX-vol shows only a significant increase after learning walks under natural skylight or a rotating linear polarizer. See text for further details. Based on data from (Grob et al. 2017, 2022)

The study by Grob et al. (2017) had shown that the ants were still able to perform the turn back towards the nest entrance behavior while pirouetting during learning walks when UV light and the position of the sun were blocked (Grob et al. 2017). The structural changes in the volume and/or numbers of synaptic complexes in the CX and MB visual pathways were used as an experimental readout to quantify the effects of different experimental conditions. The resulting structural plasticity in the ants’ brains was quite distinct between passive light exposure and following visual experience during learning walks (Stieb et al. 2010; Grob et al. 2017, 2022) (Figs. 3 and 4).

In the bulbs of the CX pathway, the number of microglomerular synaptic complexes remained unchanged following all conditions that had been applied during learning walks. As the total number of synaptic complexes in the bulbs was already high compared to dark-kept naïve ants, this supports the idea that plasticity in bulb synaptic complexes occurs before the performance of learning walks (Fig. 3). This most likely happens when the ants approach the nest entrance for the first time and while performing digging walks close to the nest entrance. Most importantly, the volumes of the CX and MB calyx including the numbers of microglomeruli in the MB calyx collar increased, but only when the ants performed learning walks under natural skylight (Grob et al. 2017, 2022) (Fig. 4). The brains of ants that had been excluded from UV light or that had performed learning walks under a static linear polarization pattern were not different from those that had remained in constant darkness or had been treated by light exposure without the performance of learning walks. This suggests that learning-walk dependent plasticity requires the active performance of learning-walk behavior and, at the same time, access to natural skylight. A more sophisticated follow-up experiment narrowed down the most relevant skylight cue (Grob et al. 2022). In the experiment, the ants performed learning walks under a special setup mounted above the nest entrance comprising an automatically rotating linear polarizer. The results show that only ants that had experienced a rotating UV sky-polarization pattern responded with a volume increase in the CX and an increase of microglomeruli in the MB calyx collar (Fig. 4). Interestingly, a rotating linear polarizer was sufficient to induce this effect. Most importantly, already a single 90° rotation per day was sufficient to trigger structural plasticity in both CX and MB circuits. An hourly rotation by 45° increased this effect, but both the linear rotation by 15°/h or unrestricted exposure to the natural skylight did not lead to any significant further increase of neuroplastic changes. Most interestingly, under all experimental conditions (except for the dark kept control group) the ants were able to perceive the natural course of the sun (as a celestial body) across the sky. This means that the rotating sky-polarization pattern is the necessary and most important cue for inducing learning-walk related neuroplasticity in both the CX and MB pathways. The moving sun alone is not sufficient to induce this effect. As could be expected, under all conditions the number of bulb synapses in the CX pathway remained unchanged after learning walks (not shown) (Grob et al. 2022).

An interesting outcome of the experiments described above was that neuroplasticity triggered by a dynamic sky-polarization pattern did also occur in the MBs (Grob et al. 2022) (Fig. 4). It seems likely that UV information is transferred to the MBs, but this has not yet been shown for *Cataglyphis* ants. In the honeybee, for example, MB output neurons show a sensitive response to UV light (Schmalz et al. 2022; Strube-Bloss and Rössler 2018). However, it is not known whether the MBs receive any direct input from polarization-sensitive neurons. The results on plasticity in the MB collar mediated by a rotating polarization pattern indicate that information about sky polarization must somehow be relayed to the MBs, whether via a direct or indirect route needs to be explored in the future.

Another important aspect is that *Cataglyphis* ants perform several learning walks over at least two days (Fleischmann et al. 2016, 2017; Freas et al. 2019). This period correlates well with the time required to induce structural neuronal changes in high-order sensory integration centers. This is also the case for structural synaptic plasticity in the MB calyx associated with the formation of stable long-term memories after associative learning, which, for example, has been shown for both long-term appetitive olfactory memory in the honeybee (Hourcade et al. 2010; Scholl et al. 2015) and for long-term aversive olfactory memory in leaf cutting ants (Falibene et al. 2015). In the same line, light-exposure related (most likely homeostatic) structural neuroplasticity in the MB pathway of both the honeybee and ant requires at least three days of light exposure (Scholl et al. 2014; Stieb et al. 2012). Both associative and non-associative plasticity related structural neuronal changes almost certainly involve protein-synthesis dependent processes and the necessary activation of gene expression and subsequent protein synthesis is likely to involve a minimum of 24 h (Becker et al. 2016).

If we combine the results on the effects of a rotating sky polarization pattern with the known necessity for the experience of the earth’s magnetic field for path integration and goal-directed views during learning walks (Fleischmann et al. 2018b), we can conclude that both geostable compass information from the earth’s magnetic field together with a rotating UV sky-polarization pattern are necessary prerequisites to induce structural neuroplasticity in the CX and MB during initial learning walks. Considering the earlier results on the role of initial learning walks and re-learning walks in memorizing the panoramic scenery (Deeti and Cheng 2021; Fleischmann et al. 2016, 2018a; Jayatilaka et al. 2018; Müller and Wehner 2010; Wystrach et al. 2014a, b), this means that sensory reception of the earth’s magnetic field, access to the panoramic scenery, and the experience of a dynamic (rotating) sky-polarization pattern over a minimum period of two days and with a minimum distance of 0.5 m are necessary for successful learning walks. Although neuroplasticity in both the CX and MB strongly suggests that the experience of a dynamic sky-polarization pattern during learning walks is crucial for acquiring or calibrating an internal representation of the solar ephemeris, this hypothesis still needs further behavioral and functional proof.

## Conclusions, open questions, and outlook

The results from visual manipulation studies during learning walks unambiguously demonstrate the role of celestial input, particularly a dynamic sky-polarization pattern, for inducing learning-related rewiring of visual neuronal circuits in the CX and MB. In addition, synaptic changes in the CX and MB pathways of naïve ants also occur after passive light exposure, most likely reflecting homeostatic plasticity before the start of learning walks. In the MBs, this form of plasticity can still be induced in old ants that have been kept in darkness. As the earth’s magnetic field is an important compass cue for goal-directed views during initial learning walks, the most obvious follow-up experiments will be long-term manipulations of the magnetic field during learning walks under natural skylight conditions and their consequences for neuroplastic calibrations in the visual pathways. The most likely outcome of this experiment is that plastic changes are absent when the magnetic field is eliminated or strongly disturbed and, therefore, not available as a compass reference during learning walks.

Many other questions and follow-up studies come to mind. For example, what is the sensory mechanism for magnetoreception in *Cataglyphis*? A recent review (Fleischmann et al. 2020b) has elaborated extensively on this question and put forward arguments favoring a magnetic particle-based mechanism that would enable light-independent and polarity-sensitive perception of the earth’s magnetic field. A study on the honeybee (Lambinet et al. 2017) provided evidence for polarity-sensitive (most likely magnetic-particle based) magnetoreception, at least in an artificial experimental setup. Systematic manipulation experiments in *Cataglyphis* using a 3D Helmholtz coil system can now test this hypothesis in the natural habitat using high-speed video analyses of nest-directed views during learning-walk pirouettes as a readout.

Where are the magnetoreceptors located, and what is the neuronal pathway mediating magnetosensation? Where in the brain does the information converge with visual input? Several lines of evidence have promoted the idea that the ants’ antennae might be sites for magnetoreception (de Oliveira et al. 2010; Fleischmann et al. 2020b; Wajnberg et al. 2010). The JO is a potential candidate and has recently been studied anatomically—both the peripheral structures in the receptor organ and afferent projections in the ant brain (Grob et al. 2021a). Each JO scolopidium comprises three receptor neurons, and sensory afferents from the JO in *Cataglyphis* converge with visual input from the ocelli and the optic lobes in posterior brain regions, the ventrolateral protocerebrum, ventral complex, saddle, and posterior slope (Grob et al. 2021a; Habenstein et al. 2020). Future tracking of antennal movements during magnetic manipulation experiments may give further hints to the potential role of the antennae in magnetoreception (Fleischmann et al. 2020b).

Further important questions come up regarding the kind of navigational information that is acquired during learning walks and how subsequent navigational performance is disturbed by visual and magnetic manipulations during learning walks. What are the behavioral consequences after manipulations of specific sky compass cues and/or the magnetic field during learning walks concerning view-based spatial orientation and the precision in the calibration of the internal representation of the solar ephemeris in foragers? Some of the required behavioral experiments are straightforward, but others are more difficult as they require follow-up studies on individually marked ants and/or experiments employing several experimental groups under the same treatment and with the necessary controls for parallel behavioral and neurobiological analyses.

It will be highly interesting and important, but also most difficult, to test whether ants experiencing manipulations during learning walks that prevent structural neuroplasticity in the CX and MB also fail in navigational tasks that require a time-compensated skylight compass. *Cataglyphis fortis* inhabits featureless environments in North African salt flats without or with less prominent landmarks or panoramic sceneries. The accessibility of “test fields” devoid of a prominent visual panorama in this environment provides ideal conditions for performing experiments to answer this question. It is very interesting in this context that previous high-resolution analyses of learning walk behavior in closely related *C. fortis, C. nodus, and C. aenescens* have shown that only *C. fortis* does not perform pirouettes (rotations on the spot about the vertical body axis including stops with nest-directed gazes) during learning walks (Fleischmann et al. 2017). Unlike the other two *Cataglyphis* species, *C. fortis* exclusively performs voltes—small, walked circles, with only rare and less distinct stops—but not pirouettes. This fuels the hypothesis that pirouettes with goal-directed stops and views are important for panoramic snapshot learning, whereas voltes might serve celestial compass calibration. Ants in cluttered habitats would then be able to efficiently perform both tasks at the same time—a collection of panoramic views, and calibration of the celestial compass—by performing two distinct behavioral routines for actively exploring both sets of visual parameters. However, close quantitative comparisons with similar body rotations that have been observed in other ant species (e.g., Deeti and Cheng 2021; Jayatilaka et al. 2018; Müller and Wehner 2010; Wystrach et al. 2014a, b) are needed to substantiate this hypothesis and to clarify the role of different types of body rotations during learning walks. Interestingly, dung beetles occasionally perform rotational movements on their dung ball to acquire celestial snapshots for strait-line orientation (el Jundi et al. 2016). This implies that the employment of body rotations for visual snapshot learning and skylight compass orientation may be based on behavioral routines that are conserved over phylogenetically large scales.

Do additional sensory modalities affect learning-walk experience? From the behavioral experiments and analyses of neuroplasticity described above, we can conclude that learning walks at least require the multisensory experience of dynamic sky polarization cues, magnetosensory information, and (if available) panoramic cues. Previous studies have shown that a minimum requirement of time (at least 2 days) and space (at least 0.5 m distance from the nest entrance) are required to learn the panoramic scenery around the nest (Fleischmann et al. 2016, 2018a). However, in addition to vision and magnetoreception, we cannot exclude that input from other sensory modalities come into play during initial learning walks. In *Cataglyphis*, olfactory orientation, especially in response to CO_2_, but also other odorants, is important during the final nest approach after homing and for exploring olfactory landscapes close to the nest site, which is combined with visual information (Buehlmann et al. 2012, 2014; Steck et al. 2009, 2011). Furthermore, when the ants screen the desert for finding food, they can form long-lasting olfactory memories for a substantial number of different odorants (Huber and Knaden 2018). Ants of the genus *Cataglyphis* possess well developed antennal lobes containing more than 200 olfactory glomeruli, the functional units for odorant processing (Grob et al. 2021b; Stieb et al. 2011). Based on this, it is conceivable that the ants also explore the olfactory landscape around the nest during their initial learning walks. Finally, wind orientation and the neuronal underpinnings of a wind compass have been explored in *Cataglyphis* and, for example, in the dung beetle and *Drosophila* (Dacke et al. 2019; Okubo et al. 2020; Wehner 2020). In some desert habitats, like North African salt flats, wind direction can be quite constant or even predictable, at least over some time periods (Wehner 2020). Much like in *Drosophila*, the JO in the antenna is likely to play an important role in using wind direction as a compass cue in *Cataglyphis* (Grob et al. 2021a). This is also supported by the demonstration of the transformation of wind perception to celestial compass coordinates (Wystrach and Schwarz 2013). Future studies are needed to characterize the importance of olfactory and mechanosensory modalities during initial learning walks.

Input from the sky compass requires time compensation, most likely by linking an internal (learned) function of the solar ephemeris to the internal clock (Wehner 2020; Wehner and Müller 1993). Neurons expressing the neuropeptide Pigment-Dispersing Factor (PDF) play important roles in neuronal clock networks of *Drosophila* and are potential candidates relaying day-time information to neuronal processing centers involved in visual navigation (Helfrich-Förster 2005; Helfrich-Förster et al. 2020). A recent study revealed a broad distribution of PDF neurons in the honeybee brain and suggested their potential role in circadian rhythmicity (Beer et al. 2018). Another study on the honeybee has identified the optic lobes as a potential site of modulatory input from PDF neurons (Zeller et al. 2015). It will be highly interesting for future studies to investigate PDF neurons and the presence of clock-related neurons in the *Cataglyphis* brain, particularly their association with visual processing and integration centers. In principle, time compensation via modulatory influences from the endogenous circadian clock could be implemented both upstream or downstream the CX and MB.

## Working model for learning-walk related multisensory input and neuroplasticity

**Fig. 5:**
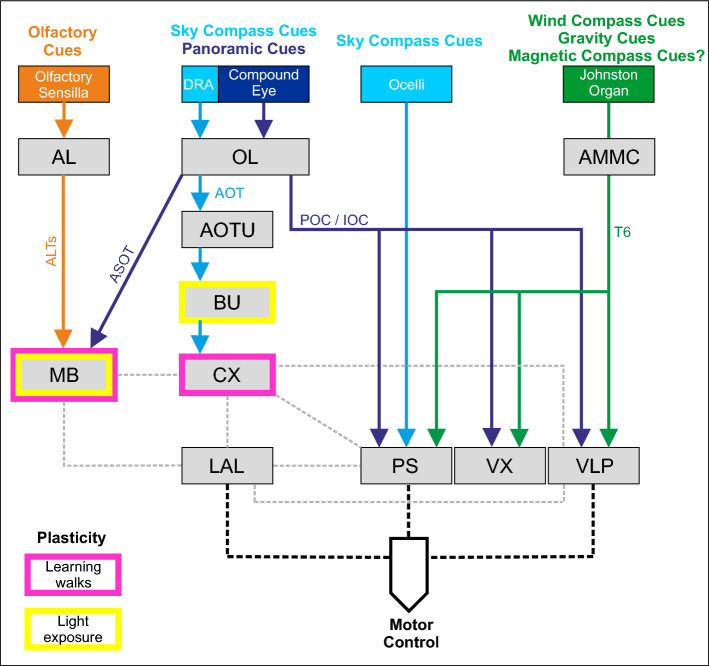
Working model for learning-walk related multisensory input and neuroplasticity in the brain of *Cataglyphis* ants. Different sensory modalities involved during learning walks and the respective receptor organs are depicted color coded in the top rows. The sensory projections to brain neuropils (shown as boxes) are drawn as arrows in the same colors. Labels show the abbreviated names of sensory tracts. The types of plasticity shown for the target neuropils of the two major visual tracts (BU, CX, MB) are color coded, where yellow depicts plasticity related to passive light exposure and magenta plasticity related to learning walks (explanatory box lower left). Solid lines indicate neuroanatomically traced pathways in the *Cataglyphis* brain. Dashed gray and black lines depict connections between neuropils that have been found in tracing or connectome studies from other insects, particularly *Drosophila, Bombyx,* and in locusts. See text for further details and for detailed references to the various sources of information depicted in the model. *AL* antennal lobe, *ALT*s antennal lobe tracts, *AMMC* antennal mechanosensory and motor center, *AOT* anterior optic tract, *AOTU* anterior optic tubercle, *ASOT* anterior superior optic tract, *BU* bulb, *CO* collar, *CX* central complex, *DRA* dorsal rim area, *IOC* inferior optic commissure, *LA* lamina, *LAL* lateral accessory lobe, *LI* lip, *MB* mushroom body, *OL* optic lobe, *POC* posterior optic commissure, *PS* posterior slope, T6 Johnston's organ afferent tract 6, *VL* vertical lobe, *VLP* ventrolateral protocerebrum, *VX* ventral complex

We propose a working model for multisensory input during learning walks and related rewiring of sensory pathways in the *Cataglyphis* brain (Fig. 5). In the scheme, color-coded solid lines indicate the known sensory pathways for visual input from the compound eyes via the optic lobes and the ocelli, mechanosensory (and potentially magnetosensory) input from the JO of the antennae, and olfactory input from the antennae. In addition to connections between brain neuropils known from studies in *Cataglyphis* (Grob et al. 2017, 2021a; Habenstein et al. 2020; Schmitt et al. 2016) (solid lines), connections between brain neuropils known from other insects are included, particularly from recent connectome studies in *Drosophila*, tracing studies in moths, or preliminary results in *Cataglyphis* (in all cases indicated by dashed lines in Fig. 5). For obvious reasons, this scheme is far from being complete. Its main purpose is to provide a ground for discussions and future planning for combinations of behavioral experiments, neuroanatomical and neurophysiological analyses, modeling studies, and functional studies on neuroplasticity. This also concerns studies on multisensory convergence in brain neuropils that have not been looked at so far and analyses on rewiring of circuits during the early ontogeny of foraging behavior (either measured as volume changes or, ideally, structural synaptic plasticity in identifiable circuits). Neuroplastic changes in brain neuropils that have already been demonstrated are indicated as magenta (learning-walk related) and yellow (sensory-exposure related) borders highlighting neuropils in Fig. 5. Due to the difficult neurophysiological access (e.g. electrophysiology or functional imaging) in ants, Fig. 5 is also intended to stimulate comparative studies in *Drosophila* and other insect models or modeling studies inspired by data on behavioral and neuronal plasticity in *Cataglyphis.* In the following, we will mainly focus on convergences of neuronal pathways that are likely to play a role in neuroplasticity triggered by multisensory learning during learning walks and sensory exposure prior to learning walks.

## Connections between the MB and CX pathways

One obvious question is how information from panoramic view memories in the MBs is integrated with heading and path integration information processed in CX circuits (Seelig and Jayaraman 2015; Stone et al. 2017). Possible solutions have been proposed in models by Collett and Collett (2018), Sun et al. (2020), and Wystrach et al. (2020). This is not only important for view memories, but also for connecting olfactory memories to path integration information in the CX. Recent studies in *Drosophila*, elegantly shown by transsynaptic staining or in serial electron microscopy connectome studies, revealed that a substantial number of MB output neurons are synaptically connected with fan shaped body neurons of the CX, mainly in the superior medial protocerebrum (Hulse et al. 2021; Li et al. 2020a; Scaplen et al. 2021). This confirms earlier predictions by Collett and Collett (2018) or in a model by Hoinville and Wehner (2018) (Fig. 5, dashed line between MB and CX). MB output neurons were shown to express learning-related changes and categorize multisensory input in the honeybee, and to exhibit changes in aversive and appetitive responses to odorants after olfactory conditioning in *Drosophila*, in this case, independent of the original valence of the odorants (Hige et al. 2015; Owald and Waddell 2015; Strube-Bloss et al. 2011; Strube-Bloss and Rössler 2018). The activation of responses in ‘aversive’ and ‘attractive’ MB output neurons might modulate navigational decisions in CX circuits via direct interactions with fan-shaped body neurons. The combination of aversive and attractive MB output in ant navigation was suggested in recent behavioral analyses and modeling approaches (Le Möel & Wystrach 2020; Murray et al. 2020). How such learning-dependent modulatory interactions may influence behavioral decisions at a mechanistic level will be a most interesting focus for future investigations. In the same context, it is highly interesting that the CX fan-shaped body of *Cataglyphis* ants is innervated by a large variety of neuropeptidergic neurons (Habenstein et al. 2021a, 2021b; Schmitt et al. 2017). These neurons may convey input from further modulatory systems that mediate internal age- or status-dependent changes, and external context-dependent influences.

Studies in the honeybee have shown that more than 30% of MB output neurons are multimodal and respond to both olfactory and visual input (Strube-Bloss and Rössler 2018). This means that both modalities converge at the level of the MB output. Therefore, panoramic view memories might interact with olfactory input at the level of the MBs and, for example, lead to cross-modal interactions during learning as it has been shown for color learning and olfaction (Becker et al. 2019; Schmalz et al. 2022; Strube-Bloss and Rössler 2018). Computation of path integration information in the CX and view-based information in the MBs have recently also been implemented in models for optimal integration of navigational information (Hoinville and Wehner 2018; Sun et al. 2020). Interestingly, long-term memory after color learning was also shown to induce structural plasticity in the fan-shaped body of the central complex of *Camponotus* ants (Yilmaz et al. 2019).

In *Cataglyphis* and, for example, in the honeybee, massive visual input is relayed from the optic lobes to the collar of the MB calyx, whereas in the fly only very few direct projections from the optic lobes mediate visual input to the MB calyx (Ehmer and Gronenberg 2002; Grob et al. 2017; Habenstein et al. 2020; Li et al. 2020b; Vogt et al. 2016; Yilmaz et al. 2016, 2019). The differences in visual innervation patterns of the MB calyces between basal and higher hymenopteran species (Farris and Schulmeister 2011) and the small number of visual connections to the MB calyx in *Drosophila* can be expected to cause differences in the way how visually activated MB output neurons may affect CX processing. These circuit differences reflect different sensory ecologies between insect species. It also emphasizes the general importance of comparative studies on multimodal information processing (Thiagarajan and Sachse 2022) and the role of differences in visual pathways on visual behaviors (Ryu et al. 2022).

Like in other Hymenoptera, olfactory information in *Cataglyphis* is relayed from the antennal lobe to the MB and lateral horn via multiple antennal lobe tracts (Kirschner et al. 2006). During learning walk behavior, in addition to memorizing the panoramic scenery, *Cataglyphis* might acquire additional homing information by learning olfactory landmarks around the nest entrance. During homing, much like view-based information, olfactory memories might be relayed to the CX via olfactory MB output neurons and influence navigational decisions. The connections of MB output neurons with the fan shaped body (also termed upper unit of the central body) suggests a prominent role of this part of the CX in multisensory integration and modulation of spatial orientation.

## Convergence of visual pathways with input from Johnston’s organ

Where does information from the JO converge with visual information and how could it be relayed to the CX? Interestingly, in both the honeybee and *Cataglyphis*, projections in the posterior slope are in very close apposition with input from ocellar interneurons (Ai et al. 2007; Grob et al. 2021a). Furthermore, anatomical and behavioral evidence suggest that the ocelli mediate information about celestial compass information in desert ants (Penmetcha et al. 2019; Schwarz et al. 2011). The convergence of sensory pathways from the ocelli and JO provides further evidence that the posterior slope represents a multimodal integration center (Currier and Nagel 2020). In *Cataglyphis*, this brain region likely integrates visual input from the ocelli, mechanosensory input (wind compass, gravity) from the JO, and, although still speculative, potentially magnetosensory information from the JO (Fleischmann et al. 2020b; Grob et al. 2021a). Furthermore, projections from the optic lobes are relayed to the posterior slope, ventrolateral protocerebrum, and ventral complex via the posterior and inferior optic commissures indicating that visual information from the compound eyes, too, may converge with multisensory input from the JO (Habenstein et al. 2020). Although for *Cataglyphis* it is still an open question how information from the posterior slope and ventrolateral neuropils might be relayed to the CX, several pathways shown in *Drosophila* provide potential candidates. For example, connections of auditory circuits from the JO to the CX (Lai et al. 2012) and for wind compass information from the anterior mechanosensory and motor center to the CX (Okubo et al. 2020). Similarly, studies in the locust and *Drosophila* revealed connections between the CX and lateral accessory lobe with the posterior slope and other neuropils in the central brain (Hadeln et al. 2020; Scheffer et al. 2020). Such feedback connections in *Cataglyphis* might be relevant for plasticity in CX neuropils observed during the active performance of learning walks under a rotating sky-polarization pattern, while the ants use the earth’s magnetic field as a compass reference (Fleischmann et al. 2018b; Grob et al. 2017, 2022). Although the posterior neuropils and central brain are not as easy to access as the CX, the bulbs, or the MBs, future studies on learning-walk induced structural neuroplasticity should also analyze these brain centers. Clearly, future tracing studies are needed to anatomically characterize the connections between these neuropils including their neurotransmitter and neuromodulator systems. The *Cataglyphis* brain atlas will be a helpful tool for such experiments (Habenstein et al. 2020). Furthermore, recent studies have revealed stage-related changes in neuropeptide modulators (e.g. Corazonin, Allatostatin) in distinct neuropils or brain regions of the *Cataglyphis* brain (Habenstein et al. 2021a, 2021b). Future studies should also characterize the neurochemistry associated with multisensory interactions and analyze potential changes in neurotransmitters and neuromodulators. As an alternative approach to live imaging techniques like calcium imaging, activity- or learning-related changes in the expression of immediate early genes in relevant brain neuropils receiving convergent multisensory input may give important insights into neuronal effects of learning-walk behavior (Sommerlandt et al. 2017, 2019).

## Pathways to premotor centers

The connections between the output from the CX and MBs to premotor centers have not yet been investigated in *Cataglyphis* ants. Clearly, future tracing studies are necessary to characterize these pathways. However, as the CX circuitry appears to be highly conserved across insect taxa (Strausfeld 2012), we can at least make some predictions from the connections already known in other insects (Steinbeck et al. 2020). The CX output is mainly relayed to the lateral accessory lobe (Pfeiffer and Homberg 2014). The lateral accessory lobe, posterior slope and ventrolateral protocerebrum can be viewed as premotor control centers as they are targeted by descending neurons that relay the information to thoracic motor control centers (Namiki et al. 2018a, 2018b; Namiki and Kanzaki 2016) (dashed black lines in Fig. 5). Therefore, in addition to information that is relayed to the CX, it appears likely that input from the JO may also exert direct modulatory influences on visually mediated responses via these descending pathways, for example by modulatory interactions in the posterior slope, ventrolateral protocerebrum, or even downstream of these neuropils. These aspects require future studies at the synaptic and ultrastructural levels. There are no indications that information from the JO is relayed to the MBs, but interactions with MB output neurons may be possible at the level of their connections with downstream neuropils like the lateral accessory lobe. These open questions related to processing of multisensory input and the role of learning and memory during the performance of learning walk behavior in *Cataglyphis* ants represent a most exciting field for multidisciplinary neuroethological research aimed at elucidating the ontogeny of spatial orientation in a highly skilled insect navigator.

## Data Availability

If applicable, figures in this review paper refer to the data provided in the original research publications.
